# Designing a Human T-Lymphotropic Virus Type 1 (HTLV-I) Diagnostic Model using the Complete Blood Count

**Published:** 2013-03

**Authors:** Masoumeh Sarbaz, Omid Pournik, Leila Ghalichi, Khalil Kimiafar, Amir Reza Razavi

**Affiliations:** 1 Department of Medical Informatics, School of Medicine and Faculty Member of Health Information Technology and Medical Records Department, School of Paramedical Sciences, Mashhad Univer­sity of Medical Sciences, Mashhad, Iran; 2 Department of Medical informatics, Faculty of Medicine, Mashhad University of Medical Sciences, Mashhad, Iran and Deputy for Health, Shahid Beheshti University of Medical Sciences, Tehran, Iran; 3 Department of Epidemiology and Biostatistics, School of Public Health, Tehran University of Medical Sciences, Tehran, Iran; 4 Department of Health Information Technology and Medical Records, School of Paramedical Sciences, Mashhad University of Medical Sciences, Mashhad, Iran and Department of Health Information Management, Faculty of Health Management and Information Sciences, Tehran University of Medical Sciences, Tehran, I.R. Iran; 5 Department of Medical informatics, Faculty of Medicine, Mashhad University of Medical Sciences, Mashhad, Iran

**Keywords:** CHAID, Machine Learning, Decision Tree, HTLV-I

## Abstract

***Objective(s):*** Infection caused by Human T-Lymphotropic Virus Type 1 (HTLV-I) can be observed in some areas of Iran in form of endemic. Most of the cases are asymptomatic, and few cases progress to malignancies and neural diseases. Designing and implementing a model to screen people especially in endemic regions can help timely detection of infected people and improve the prognosis of the disease.

***Materials and Methods:*** In this study, results of the complete blood count (CBC-diff) for 599 healthy people and the patients with different types of Leukemia and HTLV-I have been examined. Modeling was made using CHAID method. The final model was carried out based on the number of white blood cells (WBC), platelets, and percentages of eosinophils.

***Results:*** The accuracy of the final model was 91%. By applying this model to the CBC-diff results of people without symptoms or miscellaneous patients in endemic regions of our country, disease carriers can be identified and referred for supplementary tests.

***Conclusion: ***With regard to the prevalence of different complications in infected people, these individuals can be identified earlier, leading to the improvement of the prognosis of this disease and the increase of the health status especially in endemic regions.

## Introduction

Human T-Lymphotropic Virus Type 1** (**HTLV-I) is a retrovirus, which can be observed in different kinds of diseases such as lymphoma, leukemia, uveitis and neural disorders (1).

So far, several areas in the world have been known as endemic regions for HTLV-I including the southwest of Japan, some parts of Africa, and Latin America as well as some regions of Khorasan Razavi province in Iran (2-5).The prevalence of this disease in northeast of Iran is about 2.12 % (6). The prevalence of HTLV-I infection among the total population of Mashhad and blood donators is 3% and 0.44%, respectively (7, 8).

The most prevalent findings of this disease are lymphadenopathy, hepatosplenomegaly, skin diseases, hypocalcaemia, and bone lesions.

Since this virus, similar to other retroviruses, attacks leucocytes, carrying the virus genetic information can play an effective role in spreading and transmitting this kind of infection (9). As this virus has an intercellular life cycle and special tendency toward T-lymphocyte cells, monocytes, fibroblasts, and synovial cells, the transferring of this virus and disease is also carried out through infected cells (10). HTLV-I is mostly spread through the prepared blood cellular products, the tainted syringe needles, sexual intercourses, and from mothers to children by breast feeding. Mother’s milk may contain infected lymphocytes which have an important role in transferring the disease to the child (11).

The possibility of infection with this disease among people infected with T cell lymphoma leukemia is almost 1%; consequently, the infected people mostly remain as carriers of the virus. Most of HTLV-I carriers lack any symptoms; just among a few of them neural and blood symptoms may be observed (9). The effects of this disease emerge in long term. The preliminary diagnosis of this disease using blood samples can be done by using ELISA tests and confirmed by Western Blot test. Due to partly low incidence of this disease, its diagnosis is delayed in many cases that subsequently impose high costs on the patients and health organizations.

Due to its chronic and offensive nature, screening the blood donators and mothers in regard to the existence of any antibody against HTLV-I can be an effective preventive measure in endemic regions (11). Considering the limited number of patients in these two groups, we can say that limiting blood screening to these cases ends in not discovering a high proportion of patients.

Health care environment contains huge amount of data and information, but lacks enough practical information. In health care systems and laboratories, valuable data is available (12). Data mining via different methods such as interpreting complicate diagnostic tests, analyzing data from different sources (pictures, clinical data, and video clips) can provide the required support for differential diagnosis and prognosis that help clinicians to detect hidden patterns and connections in the clinical data (13).

With regard to the importance and burden of the HTLV-I infection and the limitations of their diagnosis, by using data mining techniques, we can analyze basic laboratory data of infected people in order to obtain valuable outputs for health care organizations. In this study, we intend to suggest a model for screening HTLV-I not confined to pregnant women and blood donators, but including all individuals who undergo complete blood count examination. The suggested model, obtained through mining blood examination, can be used for screening the infected people for supplementary examinations.

## Materials and Methods

In this study, one of the standards in the scope of data mining called CRISP-DM (Cross Industry Standard Process for Data Mining) is used. This standard defines inputs, outputs and the general strategies used in every stage (14).

The CRISP-DM method has been elaborated in a hierarchical model. Based on this model, the life cycle of each data mining project includes six stages. The sequence of these phases is not restricted and moving forward or backward among different phases is always required (15).

*The first phase – business understanding*

As already discussed, this disease is highly prevalent in some areas of our country, which can be seen as endemic. The detection of the virus and proper action for the involved cases can improve prevention and treatment. Furthermore, applying data mining techniques to the data can produce predicting models to achieve applicable solutions with proper efficiency (16).

In fact, predicting data mining refers to the analysis of data sets originated from lists or cases. Each case includes a number of attributes (predicting variables, explanatory variables, features or factors) and a specific variable, which is called response variable. In general, the function of predicting data mining is finding the best model which relates the features to the results. The predicting data mining aims at creating a model, which can provide reliable predictions for doctors in their prognosis and assist them in diagnosis or treatment (17). Therefore, we intend to offer a quick screening model to identify the people infected by this virus through using the data, that can be gathered easily and with the least expenditures.

*The second stage- data understanding*

In this study, the data related to 599 complete blood count tests with differentiation (CBC-diff) was collected. The results of 246 examinations were from HTLV-I patients, 142 examinations from ALL patients, 110 examinations from ATL patients and 101 examinations were taken from healthy people. These examinations were related to 107 people infected with HTLV-I, 50 patients with ALL, 44 patients suffering from ATL and 101healthy persons. The CBC of ALL and ATL patient was acquired before hospitalization and treatment. Samples were collected between 2010 and 2011.

The results for the healthy people examinations were extracted from a medical diagnostic laboratory. The test results of the patients were derived from Ghaem and Imam Reza hospital information systems in Mashhad, Iran.

*Third stage-data preparation*

In this phase of work, the data was examined with regard to its quality. The defects and shortage of data were compensated with the data existing in the reference bank. It is notable that these cases were observed in less than 2% of the cases.

Missing data was less than 1% in all fields. Also acceptable outliers, after being reviewed, and corrected if necessary, were replaced by using coercing technique for treatment of outliers.

In this stage, a new variable with binary state that specified presence or lack of infection with HTLV-I was created to be used as the target variable.

*Fourth stage –modeling*

Modeling was conducted using CHAID (Chi-squared Automatic Interaction Detection) method and version 12 of Clementine software.

The target variable used in modeling was infection with HTLV-I, which was tested by true/false samples. CHAID is a decision tree representation model which evaluates the associations between variables through exploratory analysis methods. 

CHAID method is the abbreviation of Chi-squared Automatic Interaction Detector, which is one of the decision tree methods based on Bonferroni testing (18).

*Fifth stage-model evaluation*

The existing data can be divided into two halves based on the target variable while preserving the ratio among the clusters. The first half of the data was used for the training and the second half was applied as the testing data. The designed model was optimally modified.

*Sixth stage-model dDeployment*

Model development and choice between the models were made based on the study goal, which was developing a highly sensitive model.

## Results

The data used in this study included the result of CBC-diff examinations carried out on 302 cases, among whom 94 persons were infected with leukemia, 101 cases were normal and 107 people were infected with HTLV-I. Variables were WBC and RBC count, hemoglobin, hematocrit, MCV, MCH, MCHC, platelet count, the percentage of PMN, lymphocytes and eosinophil.

The results of preliminary data analysis with ANOVA technique and the distribution of mentioned variables Modeling via CHIAD (Alpha for Splitting=0.05, Alpha for Merging=0.05) and Chi2 by applying Pearson technique was done for target variable classification.

**Table 1:** The average of variables in different groups, and significant levels of differences among these groups based on variance analysis

	HTLV-I	Leukemia	Healthy	*P*- value
WBC (10000)	13.7	66.2	8.7	0.698
RBC	4.0	3.6	4.6	<0.001
Hgb	11.6	9.8	13.2	<0.001
MCV	88.7	89.7	85.1	0.002
MCH	29.2	42.3	28.9	0.366
MCHC	34.0	32.9	33.9	0.797
Platelet	178	1009	292	0.286
Lymphocyte	29.3	27.8	32.4	0.142
Monocyte	4.7	5.4	6.2	0.504
Eosinophil	3.6	4.8	2.7	0.074

**Figure 1:**
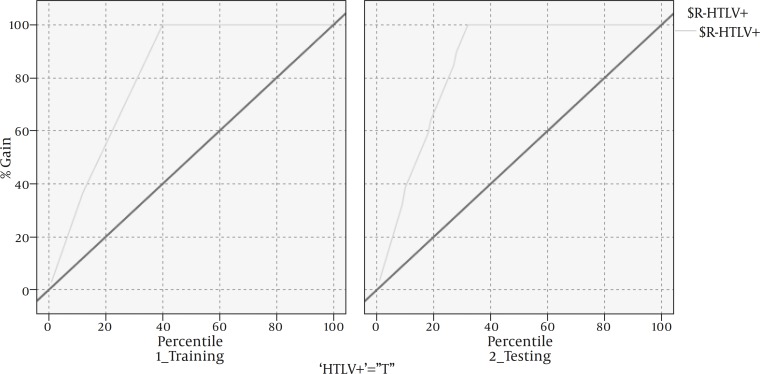
The evaluation of model performance (Gain chart

The group separation or the growth of decision tree was continued until just one percent of the cases remained in the parent branch. The final model had 91% accuracy based on area under curve (AUC) as shown in Figure 1.

**Figure 2:**
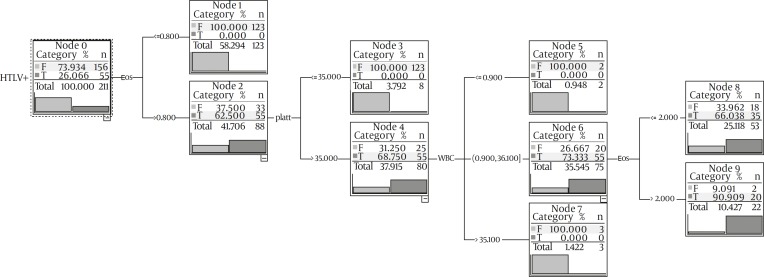
The final model obtained in terms of decision tree

The variables in the number of WBC and platelets, and the percentage of eosinophils are applied, and the final decision tree had 4 levels (tree depth=4) (Figure2).

This study indicated the accuracy of 91% and sensitivity of 95.8% in recognition of patients.

## Discussion

In this study, the final model had 91% accuracy and 95.8% sensitivity in terms of the HTLV-Iinfected individuals’ identification. This case can only be realized by just having access to information existing in a simple test of CBC-diff. Therefore, this model can be easily used to identify such patients and introduce them to conduct supplementary tests.

This model is formed based on CHAID method, which is one of the methods relying on decision tree. The general advantage of this method lies in its simple demonstration of all procedures of classification, and the possibility to extract simple rules from the resulting tree. This fact has made the application of this model possible with regard to hospitalized patients and even identifying the referred patients without hospitalization. This model can also be used for decision making and medical information management applications such as HIS (Hospital Information System), CIS (Clinical Information System), EHR (Electronic Health Record) or DSS (Decision Support System) in determining the proper direction to offer health services.

By implementing this model using CBC-diff pertaining to individuals without any symptoms or miscellaneous patients in the endemic regions of our country, possible infected persons can be detected and referred for supplementary tests. With regard to the possible prevalence of kinds of malignancies and neural disorders in infected individuals, detecting such cases can lead to timely diagnosis and burden reduction of the mentioned diseases and the enhancement of the health status especially in endemic regions.

It also provides the opportunity to decrease the disease transmission from asymptomatic patients which comprise the majority of infected people. The effects of this measure are noticeable in the case of the burden the mentioned diseases.

This study has some limitations such as not investigating some of the possible confounding factors that could have improved the model. It should be mentioned that, the quality and accuracy of the model can be improved using larger data sets. The model can be rationalized and applied in clinical practice.

## Conclusion

Decision making models based on data mining and especially the applied techniques in this article can assist clinicians in timely identification of the patients infected with HTLV-1.
